# Integrating Transcriptomic and GC-MS Metabolomic Analysis to Characterize Color and Aroma Formation during Tepal Development in *Lycoris longituba*

**DOI:** 10.3390/plants8030053

**Published:** 2019-02-28

**Authors:** Yuanzheng Yue, Jiawei Liu, Tingting Shi, Min Chen, Ya Li, Juhua Du, Haiyan Jiang, Xiulian Yang, Huirong Hu, Lianggui Wang

**Affiliations:** 1 Key Laboratory of Landscape Architecture, Jiangsu Province, College of Landscape Architecture, Nanjing Forestry University, Nanjing 210037, China; yueyuanzheng@njfu.edu.cn (Y.Y); ljw040018@sina.com (J.L.); tingtingspx@163.com (T.S.); xiaoximao2009@sina.com (M.C.); LY1885117@163.com (Y.L.); DJH2585255213@163.com (J.D.); jhy0215@foxmail.com (H.J.); yangxl339@sina.com (X.Y.); 2 Co-Innovation Center for Sustainable Forestry in Southern China, Nanjing Forestry University, Nanjing 210037, China; 3 Key Laboratory of Horticultural Plant Biology, Ministry of Education, College of Horticulture and Forestry Sciences, Huazhong Agricultural University, Wuhan 430070, China; huhuirong@mail.hzau.edu.cn

**Keywords:** *Lycoris longituba*, tepals, color fading, aroma formation, volatile organic compounds

## Abstract

*Lycoris longituba*, belonging to the Amaryllidaceae family, is a perennial bulb bearing flowers with diverse colors and fragrance. Selection of cultivars with excellent colored and scented flowers has always been the breeding aim for ornamental plants. However, the molecular mechanisms underlying color fading and aroma production during flower expansion in *L. longituba* remain unclear. Therefore, to systematically investigate these important biological phenomena, the tepals of *L. longituba* from different developmental stages were used to screen and analyze the metabolic components and relevant genes. Utilizing the Illumina platform, a total of 144,922 unigenes were obtained from the RNA-Seq libraries. Kyoto Encyclopedia of Genes and Genomes (KEGG) enrichment analysis indicated that the phenylpropanoid biosynthesis and flavonoid biosynthesis pathways might play important roles during color and aroma changes. Metabolomic analysis identified 29 volatile organic components (VOCs) from different developmental stages of *L. longituba* tepals, and orthogonal partial least-squares discriminate analysis (OPLS-DA) revealed that *trans*-β-ocimene—a terpene—was the most important aroma compound. Meanwhile, we found the content of anthocyanin was significantly reduced during the tepal color fading process. Then, we identified two *dihydroflavonol-4-reductase* (*DFR*) and three *terpene synthase* (*TPS*) genes, for which expression changes coincided with the production patterns of anthocyanins and *trans*-β-ocimene, respectively. Furthermore, a number of MYB and bHLH transcription factors (TFs) which might be involved in color- and aroma-formation were also identified in *L. longituba* tepal transcriptomes. Taken together, this is the first comprehensive report of the color and fragrance in tepals of *L. longituba* and these results could be helpful in understanding these characteristics and their regulation networks.

## 1. Introduction

*Lycoris longituba*, commonly known as Chinese tulip, is a bulbiferous species of the Amaryllidaceae family and distributed in central eastern China [1]. It can tolerate extremes of drought, waterlogging and shade, as well as poor soil conditions. Its plentiful flower colors, large tepals, elegant fragrance, and some medicinal potential make it a popular ornamental plant [2].

Flower color and fragrance are two critical factors in attracting pollinators; the various colors and particular scents are also key ornamental traits within landscape plants [3,4]. The coloration of the plant flowers is mainly attributed to the accumulation of anthocyanins that belong to a class of plant flavonoid metabolites, which are the most common pigments and best studied compounds in plants [5,6,7]. Many enzymes can catalyze anthocyanin synthesis, especially dihydroflavonol 4-reductase (DFR), which can directly increase anthocyanin accumulation and lead to the flower formation when heterologously expressed in tobacco [4]. The activity of anthocyanin biosynthesis enzymes is usually regulated by the MYB-bHLH-WD40 complex which consists of different classes of transcription factors of R2R3-MYB, basic helix–loop–helix (bHLH), and WD40-repeat [8,9]. In *Phalaenopsis* spp., *PeMYB11* could regulate variegated pigmentation of tepals by controlling the expression of anthocyanin biosynthetic genes [10]. In *Malus domestica*, *Fragaria chiloensis*, and grapevine, the *MdMYB10*, *FcMYB1*, and *VvMybPA2* have been demonstrated to regulate the accumulation of anthocyanins/proanthocyanidins of fruits [11,12,13]. Additionally, the MYB transcription factors TT2, bHLH transcription factors TT8, and the WD40 repeat protein TTG1 could also influence anthocyanin biosynthesis by regulating downstream gene expression in *Arabidopsis thaliana* [14].

Floral fragrance is made up of specialized volatile metabolites such as terpenoids, phenylpropanoids (including benzenoids), and fatty acid derivatives. The content changes of these volatile components in the blend could directly lead to distinct scents [15]. Terpenoids are the largest class of floral fragrances and have been taken as the critical members of aroma compounds [16,17]. Up to now, many *terpene synthase* genes (*TPSs*) have been isolated in various species [18,19,20,21,22,23]. Meanwhile, some scent-related transcription factors (TFs) which could regulate the expression of *TPSs* and floral scent formation have also been identified, such as *AtMYC2* in *Arabidopsis thaliana* [24], *HcMYB1* and *HcMYB2* in *Hedychium coronarium* [25], *AaNAC2*, *AaNAC3*, and *AaNAC4* in *Actinidia arguta* [26], and *CitERF71* in *Citrus sinensis* [27]. However, neither the composition of floral scent nor the scent-related gene in *L. longituba* is available.

In previous work, four critical anthocyanins in different colors of *L. longituba* tepals have been well identified from 44 floral color natural variants, and the different amounts of these anthocyanin components were demonstrated as the important determinants for the natural variation of flower colors in *L. longituba* [1]. A previous study identified 4992 ESTs of *L. longituba* from a mixed floral bud library [28]. To date, a total of 338 putative TFs were identified from three floral tissue EST libraries of *L. longituba*, which could significantly contribute to the further analysis of florescence progress [2]. However, due to technical limitations, the TFs obtained from the above research were insufficient, and the overall molecular regulation mechanisms involved in floral development still need to be investigated. It has been verified that the genome of *Lycoris* is very large (>20 Gb) [29]. In absence of a complete genome sequence, RNA-Seq technology could be taken as the most effective and economical tool for the whole genome transcriptome analysis. Until now, this strategy has been successfully used in exploring the molecular mechanism of leaf color change in *Paeonia suffruticosa* [7], fruit color change/peel color mutant in *Myrica rubra* and *Ficus carica* [30,31], petal color change/spot formation in *Prunus persica* [32], and tepal color/bicolor development in *Lilium* ‘Sorbonne’ [33]. Recently, the aroma biosynthesis regulation mechanism has also been well characterized by sequencing the flower samples in *Chimonanthus praecox* and *Osmanthus fragrans* [18,34].

*L. longituba* is a special aromatic species of *Lycoris*, and the red color of tepals will gradually shade with the expansion of flowers. In this study, a comparative transcriptomics and metabolomics analysis was carried out using the tepals from different developmental stages of *L. longituba* based on Illumina sequencing and gas chromatography-mass spectrometer (GC-MS). The critical pathways, as well as structural genes and transcription factors that related to color fading and aroma formation of *L. longituba* tepals were systematically identified, which would help to advance the knowledge and provide a more sufficient genetic resource for further exploration of the molecular regulation mechanism of tepals’ color and fragrance changes in *L. longituba*.

## 2. Results

### 2.1. Anthocyanin Level in the Different Tepal Development Stages of Lycoris longituba

**Figure 1:**
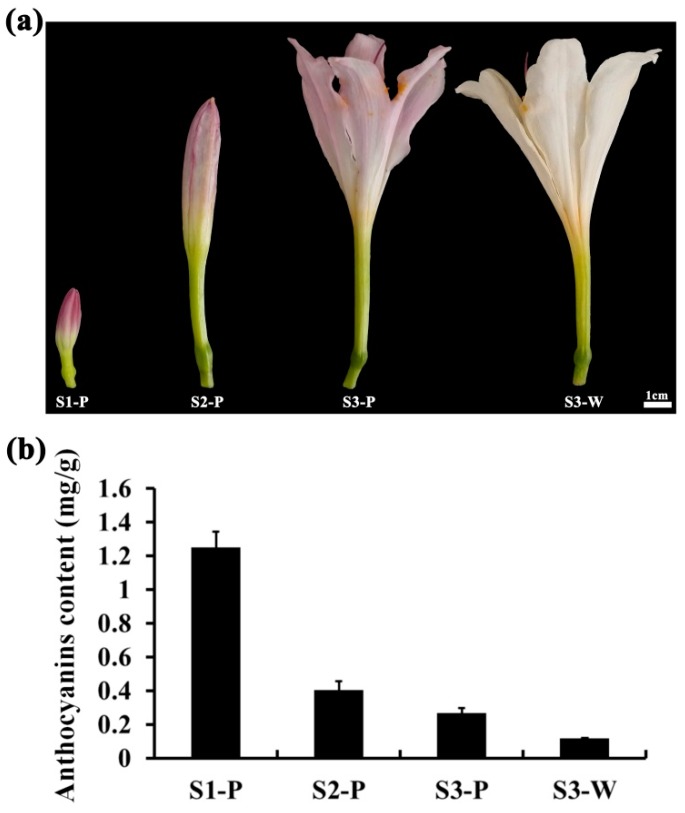
*Lycoris longituba* tepals and anthocyanin content at different samples. (**a**) Tepals of *L. longituba* ‘Pink’ and ‘White’ used for de novo transcriptome assembly (S1-P: small bud stage of *L. longituba* ‘Pink’; S2-P: medium bud stage of *L. longituba* ‘Pink’; S3-P: opening stage of *L. longituba* ‘Pink’; and S3-W: opening stage of *L. longituba* ‘White’); (**b**) content of anthocyanin in different tepal stages.

In the small bud stage, the red color of *L. longituba* ‘Pink’ tepal was very deep, and then the color intensity was significantly decreased with the rapid elongation of tepals, as shown in Figure 1a. In contrast, the tepal color of *L. longituba* ‘White’ was always white, as shown in Figure 1a. As shown in Figure 1b, the anthocyanin content in *L. longituba* ‘Pink’ was dramatically reduced from S1-P to S3-P, and nearly no anthocyanin was detected in S3-W, as shown in Figure 1b. These results suggested that content changes of anthocyanin could be the main reason that led to the tepals’ red color fading of *L. longituba* ‘Pink’.

### 2.2. Transcriptome Sequencing and de Novo Assembly

Twelve total RNA samples were isolated from different *L. longituba* tepal developmental stages S1-P, S2-P, S3-P, and S3-W. These RNA samples were at concentrations of about 200–500 ng/μL with OD260/280 ≥ 1.9 and the RNA Integrity Numbers (RINs) of 8.6–10.0 were used for cDNA library construction. The Illumina HiSeqTM 4000 platform was used to obtain the dataset of 12 cDNA libraries. About 663.25 million raw sequencing reads with a length of 150 bp were generated, and after discarding the low-quality reads, we obtained about 85.23% (565.28 million) clean reads. For all 12 samples, the quality score above 20 (Q20) was ~98.20% and the GC percentages were 45.55–46.88%. Using Trinity software, the de novo assembly totally generated 144,922 unigenes, of average length 941 bp, from the twelve tepal transcriptomes, as shown in Table 1. In this research, the N50 was determined to be 1527 bp, which indicated that the quality of sequence assembly was good. All raw high throughput sequence data have been deposited in the NCBI Sequence Reads Archive (SRA) with the accession number PRJNA490415.

### 2.3. Functional Classification of Genes during Tepal Development Stages

The assembled unigenes were annotated using blastx against NCBI nonredundant protein (Nr), Swiss-Prot, Kyoto Encyclopedia of Genes and Genomes (KEGG), and Cluster of Orthologous Groups (COG) protein sequence databases with an E-value ≤10^−5^. A total of 85,563 (59.04%) unigenes could be annotated while other unigenes had no significant BLAST hit, as shown in Table 2, indicating that numerous new genes specific to *L. longituba* are still functionally unknown and need to be further studied in the future. Remarkably, the plant species with the top three numbers of blastx hits were *Elaeis guineensis* (23,200 transcripts; 29.62%), *Phoenix dactylifera* (19,045 transcripts; 24.31%), and *Musa acuminata* (6512 transcripts; 8.31%), as shown in Table S1. These results implied that the assembled *L. longituba* transcripts shared similarity with transcripts from several monocotyledons and were reliable. Based on sequence homology, gene ontology (GO) assignment analysis was performed. Of the 85,563 annotated unigenes, 44,813 (52.37%) sequences were assigned into three main categories (biological process, cellular components, and molecular function), which could be further distributed under 58 GO terms, as shown in Figure S1. Metabolic process, cell, and catalytic activity were the most highly enriched GO terms in biological process, cellular components, and molecular function categories, respectively.

### 2.4. Identify Differentially Expressed Unigenes between Tepal Transcriptomes

**Figure 2:**
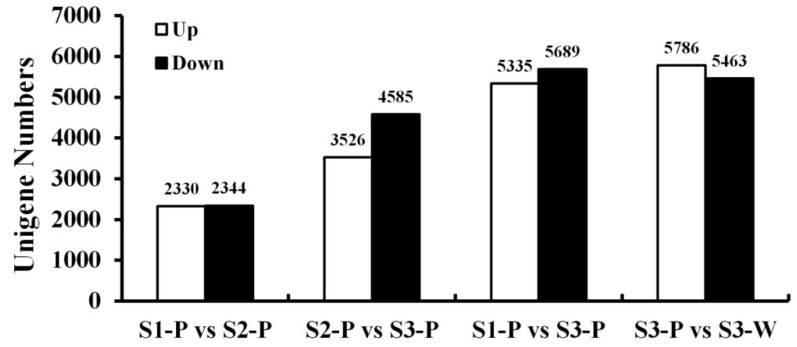
Statistics of differentially expressed unigenes (DEGs) between two different samples. White: up-regulated unigenes; black: down-regulated unigenes.

To identify differentially expressed unigenes (DEGs) during tepal color fading, the unigenes that were differentially expressed between developmental stages and opening tepals with different colors were compared and shown in Figure 2. Among the four comparisons, the smallest number of DEGs was between the S1-P and S2-P libraries (4674), of which 2330 were up-regulated and 2344 were down-regulated, and the largest number of DEGs was between the S3-P and S3-W libraries (9958), with 5786 up-regulated and 5463 down-regulated unigenes. As the tepals faded from S2-P to S3-P, 8110 unigenes were differentially expressed, with 3526 up-regulated and 4584 down-regulated unigenes. In the S1-P vs. S3-P comparison, 11,024 DEGs were detected, including 5335 up-regulated and 5689 down-regulated unigenes. The comparison between S1-P and the other tepal developmental stages (S2-P and S3-P) showed that the number of up and down-regulated unigenes were both significantly increased as the tepals developed.

### 2.5. KEGG Pathway Enrichment

According to KEGG pathway enrichment analysis (*p*-value < 0.05), the phenylpropanoid biosynthesis (96 DEGs, ko00940), flavonoid biosynthesis (58 DEGs, ko00941), flavone and flavonol biosynthesis (23 DEGs, ko00944), and anthocyanin biosynthesis (4 DEG, ko00942) pathways which related to color formation were significantly different as compared to S3-P and S3-W. Interestingly, except for the anthocyanin biosynthesis and flavonol biosynthesis pathways, the phenylpropanoid biosynthesis and flavonoid biosynthesis pathways were also identified in the S1-P vs. S2-P, S2-P vs. S3-P, and S1-P vs. S3-P comparisons. These results suggested that the phenylpropanoid biosynthesis and flavonoid biosynthesis pathways could play critical roles during red color fading and aroma formation. The statistically enriched pathways between each two transcriptomes are shown in Figure S2.

### 2.6. Validation of the Gene Expression Profiles by qRT-PCR

**Figure 3:**
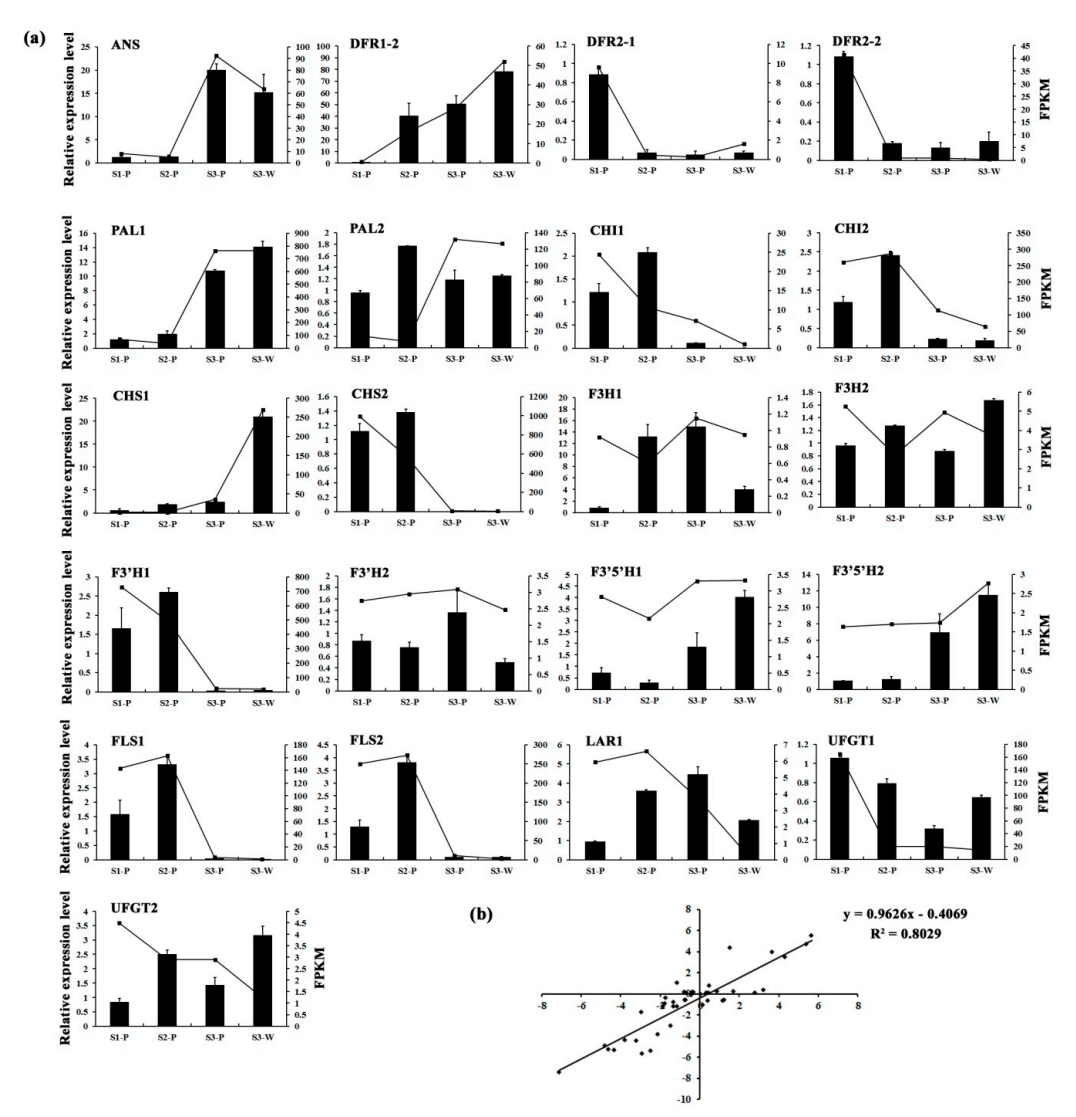
The qRT-PCR validation of DEGs. (**a**) The relative expression levels of 21 pigmentation-related candidate unigenes. The left *y*-axis denotes the relative transcript amount obtained by qRT-PCR. The right *y*-axis represents the fragments per kilobase per million fragments (FPKM) value of each gene using RNA-Seq analysis. Error bars indicate the standard errors. (**b**) Correlation analysis of the gene expression value from RNA-Seq and qRT-PCR.

To validate the transcription profile revealed by RNA-Seq data, the expression levels of 21 genes from the flavonoid biosynthesis pathway were also assessed using qRT-PCR, as shown in Figure 3a. Linear regression analysis was used to obtain the overall correlation coefficient between RNA-Seq and qRT-PCR data, which showed a good correlation (*R* = 0.89) between these two data, as shown in Figure 3b, indicating that the 12 transcriptomics data were reliable.

### 2.7. Metabolome Analysis of Lycoris longituba Tepal Development Stages by GC-MS

**Figure 4:**
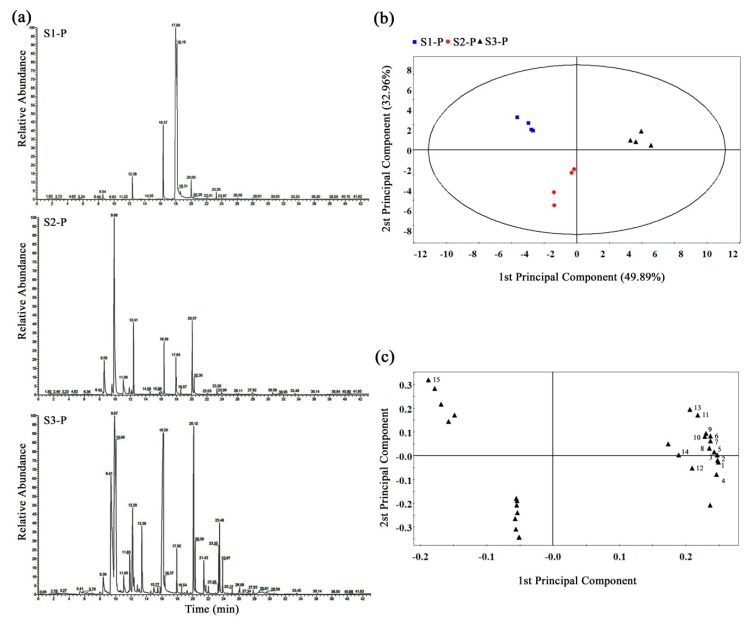
GC-MS metabolomic analysis of *L. longituba* tepals. (**a**) Typical chromatograms of *L. longituba* tepals under S1, S2, and S3. (**b**) Score plot and (**c**) loading plot of tepal GC-MS profiles of *L. longituba* according to different developmental stages using orthogonal partial least-squares discriminate analysis (OPLS-DA). Top 15 metabolites coded in the loading plot are: (1) caryophyllene, (2) *trans*-β-ocimene, (3) benzoic acid, methyl ester, (4) hexadecane, (5) benzoic acid, 2-phenylethyl ester, (6) β-myrcene, (7) butyl aldoxime, 3-methyl-, (8) 3-methoxy-5-methylphenol, (9) 1-butanol, 3-methyl-, benzoate, (10) benzyl nitrile, (11) benzene, (3-nitropropyl)-, (12) benzenepropanoic acid, α-(hydroxyimino)-, (13) 2,4,6-octatriene, 2,6-dimethyl-, (E,Z)-, (14) 1,6,10-dodecatrien-3-ol, 3,7,11-trimethyl-, (E)-, (15) octanoic acid, methyl ester.

To investigate the volatile metabolic components changes of *L***.** *longituba* tepals in the opening processes, we obtained the GC-MS total ion current (TIC) chromatograms for nine *L. longituba* tepal samples from three typical developmental stages (S1-P, S2-P, and S3-P), as shown in Figure 1a. The obvious differences of chromatographic peaks were observed between sample groups, and the retention times were fairly consistent and reproducible, as shown in Figure 4a. In this study, a total of 29 metabolites were identified in our sample libraries across all samples, as shown in Table 3.

To assess the volatile components profile changes during *L. longituba* tepal development, the orthogonal partial least-squares discriminate analysis (OPLS-DA) plot was generated from the GC-MS metabolite data of S1-P, S2-P, and S3-P tepals and showed clear metabolic differences between two stages. Remarkably, S1-P, S2-P, and S3-P tepals could be completely separated sufficiently by use of two principal components. The first principle component (PC1, accounting for 49.89%) and the second component (PC2, accounting for 32.96%) of the variation in the data could separate all three types of tepals with no outliers, as shown in Figure 4b. The contribution of each variable to PC1 and PC2 was also calculated by giving each variable a weight value. The top two core differential metabolites of PC1 and PC2 discrimination were caryophyllene and *trans*-β-ocimene, as shown in Figure 4c. Interestingly, the *trans*-β-ocimene, which had a high content in S3-P, was not detected in S1-P and S2-P, as shown in Table 3.

### 2.8. Analysis of Candidate Genes Related to Color and Fragrance Metabolics

**Figure 5:**
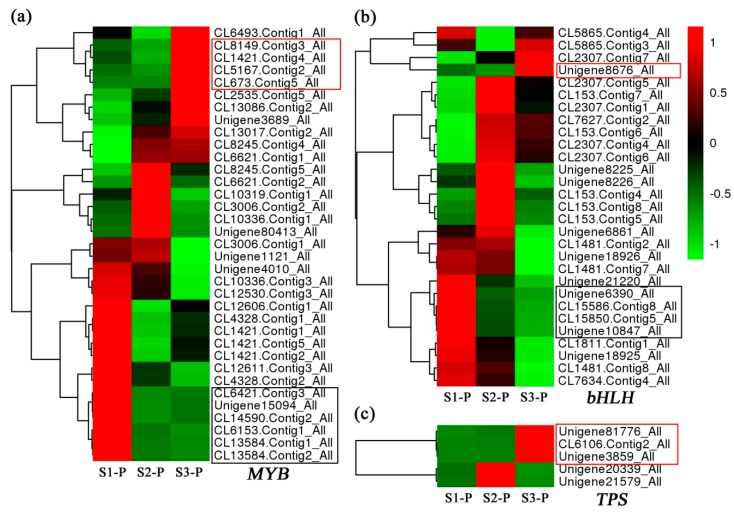
The expression profiles of differential expressed candidate genes. The heatmaps are generated according to the average expression levels of *MYB* (**a**), *bHLH* (**b**), and *TPS* (**c**) genes based on log2 transformed FPKM data. Green means low expression, and red means high expression.

To explore the genetic regulation of *L. longituba* tepal color fading and aroma emission, the genes which have been reported to be involved in these two metabolic pathways were selected. With the development of tepals, several anthocyanin biosynthesis structural genes had the lowest expression levels in S3-P, such as the DFRs, CHIs, CHS2, F3’H1, and FLSs, as shown in Figure 3a. Especially, the DFR-annotated unigenes (*DFR2-1* and *DFR2-2*) which had more than a 7-fold down-regulated expression level in S1-P vs. S3-P, as shown in Figure 3a. Five *TPS* genes were also identified from the DEGs, and interestingly three (*Unigene81776*, *CL6106.Contig2*, and *Unigene3859*) of them were predominantly expressed in S3-P, as shown in Figure 5c.

The spatial and temporal expression of pigment structural and aroma genes were usually controlled by transcription factors from MYB and bHLH [14,25]. In this study, 35 MYBs and 29 bHLHs with fragments per kilobase per million fragments (FPKM) ≥ 5 were identified from DEGs, as shown in Figure 5a,b. Among them, six MYBs and four bHLHs (in black frames) had the similar down-regulated expression trends with *DFR2-1* and *DFR2-2*, and four MYBs and one bHLH (in red frames) had the similar up-regulated expression patterns with the above three *TPSs*, as shown in Figure 5.

## 3. Discussion

Flower color and fragrance are considered to be two critical characters that influence plants’ ornamental value and insect pollination. The pigmentation and aroma formation processes involve multiple gene expression networks and complex biochemical pathways. However, the underlying molecular regulation mechanism of flower color fading and fragrance synthesis in *L. longituba* remains to be uncovered. To date, transcriptome sequencing technology has been widely used in exploring the critical metabolic pathways and functional genes that are involved in color biosynthesis and aroma formation in various species, even those that lack reference genomes. In this study, the tepal transcriptomes in small bud, medium bud, and opening stages of *L. longituba* were compared. The phenylpropanoid biosynthesis, flavonoid biosynthesis, and flavone and flavonol biosynthesis pathways which related to the color and aroma formation were highlighted between each two transcriptomes by KEGG pathway enrichment analysis, as shown in Figure S2. This result implied that these pathways could play critical roles in the two ornamental characters formation in *L. longituba* tepals.

It has been demonstrated that the different content of anthocyanidin was a critical reason for red color alteration in plant organs [9,35]. In this research, tepal color was altered from deep red to pink accompanying *L. longituba* flower bud development, as shown in Figure 1a. Remarkably, the anthocyanidin level of the tepal was significantly reduced during the color fading processes, as shown in Figure 1b. This result suggested that the decreasing of anthocyanidin accumulation could be the directly responsible for the color fading of *L. longituba*.

Dihydroflavonol-4-reductase (DFR), which is a downstream enzyme in anthocyanin biosynthesis, could regulate the critical rate-limiting step of anthocyanin biosynthesis processes by catalyzing dihydroflavonols to leucoanthocyanidins [4,36]. Recently, a series of *DFR* gene homologs have been identified in various plant species such as in *Rosa rugosa* [4], *Camellia sinensis* [37], *Populus trichocarpa* [38], and *Calibrachoa hybrida* [39]. The previous works have confirmed that overexpression of *DFR* genes within tobacco and petunia could accelerate the anthocyanin accumulation and promote the red coloration of flowers [4,39]. Here, two *LlDFRs* (*DFR2-1* and *DFR2-2*) were isolated from DEGs. Both of them had the highest expression levels in the S1-P stage (deep red bud), then significantly reduced in the S2-P stage (pink red bud), which were positively correlated with the content variation trend of anthocyanins in *L. longituba* tepals, as shown in Figure 1b and Figure 3a. Previous studies have demonstrated that the expressions of anthocyanin biosynthesis structural genes are controlled by the MYB, bHLH, and WD40 transcription factors [40], such as *PhAN11* in *Petunia hybrida* [41], *PeMYB11* in *Phalaenopsis* spp. [10], *MdMYB10* and *MdbHLH3* in *Malus domestica* [11,42], *CmMYB6* in *Chrysanthemum* [43], *AtMYBL2* in *Arabidopsis thaliana* [44], *FaMYB1* in *Fragaria ananassa* [45], as well as *PpMYB16* and *PpMYB111* in *Prunus persica* [46]. In this study, several *MYB* and *bHLH* genes, which had similar expression patterns with anthocyanidins and *DFR* genes, were identified in these transcriptomes, as shown in Figure 5. This evidence indicated that the down-regulated expression of *DFR* genes during the tepal developmental processes might be controlled by the above transcription factors, and these TFs could directly control the flower color fading.

Recently, GC–MS technology has been successfully used to analyze qualitative and quantitative differences in aroma-related metabolomics, and some important volatile terpenes which play dominant roles in the floral scent formation have been well demonstrated, such as in *Lilium* [47], *Chimonanthus praecox* [18], and *Osmanthus fragans* [19]. Here, a total of 29 floral volatile organic compounds (VOCs) and 6 volatile terpenes were identified from three developmental stages of *L. longituba* tepals, as shown in Figure 4. In previous studies, linalool has been verified as one of the critical aroma metabolites in various plants [18,19]. Conversely, linalool was undetected in the tepals of *L. longituba*, as shown in Table 3, which indicated that the composition of *L. longituba* floral aroma could be very particular. Interestingly, some VOCs were specifically emitted in the different stages; especially the *trans*-β-ocimene and β-myrcene which belong to the monoterpenes, which were only detected in the strongest aroma period of opening flowers, as shown in Table 3. Meanwhile, OPLS-DA loading values showed that the top two critical components were caryophyllene and *trans*-β-ocimene, as shown in Figure 4c, and the content of *trans*-β-ocimene was much higher than caryophyllene in the full-blooming floral tepals (S3-P), as shown in Table 3. Taken together, we concluded that the *trans*-β-ocimene could be the most important aroma compound during the *L. longituba* floral fragrance emission.

It has been demonstrated that the *TPSs* could control the β-ocimene biosynthesis at the transcriptional level [48,49,50]. In this study, the expression patterns of three *TPSs* were in excellent agreement with the emission trend of *trans*-β-ocimene in *L. longituba* tepals, as shown in Figure 5c and Table 3, implying that the three TPS members might directly influence the protein abundance of ocimene synthase. Some members of the MYB and bHLH families have also been confirmed that could control the floral aroma formation, such as the *CpMYC2* [18], *AtMYC2* [24], *HcMYB1*, and *HcMYB2* [25]. In this research, several *MYB* and *bHLH* genes which had the consistent expression patterns with *trans*-β-ocimene and *TPS* genes were identified, as shown in Figure 5a,b, suggesting that these genes could be involved in the regulation of aroma formation.

The color fading and aroma formation during the *L. longituba* tepal development could be two very complex and dynamic processes. While many candidate genes have been identified in this work, the functions of these members still need to be investigated in our future studies.

## 4. Materials and Methods

### 4.1. Plant Materials

Plants of *L. longituba* ‘Pink’ and ‘White’ were grown in the Lycoris Experimental Plantation of Nanjing Forestry University in Nanjing, China. The *L. longituba* tepals of three typical developmental stages were selected based on tepal length, which were S1-P (15 ± 5 mm tepals at small bud stage), S2-P (55 ± 5 mm tepals at medium bud stage), S3-P (90 ± 5 mm tepals at opening stage) of ‘Pink’, and S3-W (90 ± 5 mm tepals at opening stage) of ‘White’, as shown in Figure 1a. At each stage, the fresh samples of tepals were harvested and immediately frozen in liquid nitrogen and stored at -80 ºC.

### 4.2. Anthocyanin Level Measurement

Freeze-dried tepals were finely ground and 0.2 g was extracted with 2 mL acidic methanol (0.1% hydrochloric acid) at 4 ºC in darkness for 12 h, mixing the extract up and down every 6 h. Then, the extract was centrifuged at 12,000 rpm for 10 min, the supernatant was diluted 4 times with acidic methanol, and the absorbances were tested spectrophotometrically at 530 and 657 nm. Finally, total anthocyanin was defined using the equation: Q = (A_530_ − 0.25 × A_657_) × FW − 1, where Q = total anthocyanins; A_530_ = absorption at 530 nm; A_657_ = absorption at 657 nm; FW = fresh weight of tepals (g). Three biological replicates were performed for each group.

### 4.3. RNA-Seq and de Novo Assembly

Total RNA from three biological replicates was independently isolated from the tepals of *L. longituba* using RNAiso Reagent (Takara, Otsu, Japan) according to the previously described method [51]. The RIN value of total RNA sample was examined with Agilent 2100 Bioanalyzer (Agilent Technologies, Palo Alto, CA, USA), and the concentration was assessed using NanoDrop (Thermo Scientific, Waltham, MA, USA).

Twelve cDNA libraries which consisted of separate RNA samples from *L. longituba* ‘Pink’ tepals of three different developmental stages (S1-P, S2-P, and S3-P) and *L. longituba* ‘White’ tepals (S3-W) were prepared using the TruSeq RNA Sample Preparation Kit (Illumina, San Diego, CA, USA) according to the manufacturer’s instructions. Firstly, the enriched mRNA were fragmented into short fragments and reverse transcripted into first-strand cDNA. Then after second-strand cDNA synthesis, end repair, adapter ligation, and PCR amplification, the cDNA library products were sequenced on an Illumina HiSeq^TM^ 4000 (Illumina, San Diego, CA, USA) instrument using paired-end sequencing technology by staff at Gene Denovo Biotechnology Corporation (Guangzhou, China). After the raw reads removing the adapter sequences, reads with more than 20% low-quality bases (quality value <20) and ambiguous nucleotides (denoted with an “N” in the sequence trace) of the raw reads, the high-quality clean reads were accomplished assembled using Trinity software [52]. In order to avoid the interference of alternative splicing transcripts, only the longest transcript was taken as the unigene in this research.

### 4.4. Sequence Annotation and Gene Expression Difference Analysis

The assembled unigenes functional annotations were performed through a blastx search against four public protein databases including NCBI nonredundant protein (Nr), Swiss-Prot, Kyoto Encyclopedia of Genes and Genomes (KEGG), and Cluster of Orthologous Groups (COG) of proteins, with an E-value of less than 1e-5. The Blast2GO software (http://blast2go.bioinfo.cipf.es/) was used to acquire GO terms of the unigenes.

The expression level of unigenes was calculated using fragments per kilobase per million fragments mapped (FPKM) method [53]. In this study, the false discovery rate (FDR) ≤ 0.001 and the absolute value of log_2_Ratio ≥ 2 were taken as the threshold for significantly differential expression of unigenes. All differentially expressed unigenes (DEGs) were mapped to the KEGG pathway database and the numbers of unigenes for every KEGG Orthology (KO) term were calculated. Significantly enriched KO terms from the set of DEGs were identified by comparing the observed DEG count to the expected count of the genes involved in a given pathway with a random distribution of the *L. longituba* tepal transcriptome using the formula of the hypergeometric test [54].

### 4.5. qRT-PCR Analysis

Using TransScript One-Step gDNA Removal and cDNA Synthesis SuperMix kit (Transgene, Beijing, China), 2 µg of total RNA was reverse transcripted to the first-strand cDNA on the basis of the manufacturer’s instructions. The SYBR Premix Ex TaqTM Ⅱ kit (Takara) was used to perform qRT-PCR in the ABI 7500 Fast Real-Time PCR System (Applied Biosystems, Applied Biosystems, Cheshire, UK according to the manual’s description [55]. The relative expression level of genes was calculated by the 2^−ΔΔ*C*T^ method. To ensure reliability, the expression data of each gene was obtained from three independent biological replications (each biological replication included three technical replications). The data are shown as mean values ± SE (standard error). All primer pairs were designed by Primer 5 software and listed in Table S2, and the specificity of them was assessed by the sequencing of amplified qRT-PCR products. The *ELF* gene was used as an internal reference control [56].

### 4.6. GC-MS Analysis

Fresh tepals at three different stages (S1-P, S2-P, and S3-P), defined by the size of the flower as shown in Figure 1a, were picked from the plants at the same time as samples that were collected for the above transcriptome studies. Sampling was replicated four times, and the samples were quickly put into polyethylene bags impermeable to gases, kept in the ice-box, and analyzed immediately. Headspace solid phase microextraction (SPME) combined with GC-MS was used to determine the identity and quantity of the fragrance volatiles. Tepals (0.3 g) were placed in a 4 mL solid-phase microextraction vial (Supelco Inc, Bellefonte, PA, USA), 1 μL of 1000x diluted ethyl caprate (Macklin Inc, Shanghai, China) was added, and vials were capped with a 65 µm DB-5 ms extraction head (Supelco Inc). The oven temperature was programmed at 60 °C for 2 min, increasing at 5 °C/min to 150 °C, then increasing at 10 °C/min to reach 250 °C, followed by maintaining the temperature of the transfer line at 250 °C and helium was used as the carrier gas at a linear velocity of 1.0 mL/min. Mass detector conditions on MS were carried out according to our previous method [57]. The quantities of the volatile aroma compounds were calculated by normalizing the peak-areas and volatile compounds were first identified using the NIST98 database (Agilent). SIMCA-P 11.5 software (Umetrics AB, Umea, Sweden) was selected to test the differences in the metabolite levels of *L. longituba* tepals from different developmental stages by orthogonal partial least-squares discriminate analysis (OPLS-DA).

## Figures and Tables

**Table 1:** Transcriptome assembly statistics for *L. longituba*.

Description	Transcripts
Number of transcripts	144,922
Total assembled bases	136,324,908
Average length (bps)	941
N50 (bps)	1527
GC content (%)	46.11

**Table 2:** Summary of the annotations from public databases. Nr: NCBI nonredundant protein; GO: gene ontology; KEGG: Kyoto Encyclopedia of Genes and Genomes.

Database	Number of Annotated Unigenes	Percentage of Annotated Unigenes (%)
Nr	78,336	54.05
Nt	63,362	43.72
Swiss-Prot	56,768	39.17
GO	44,813	30.92
KEGG	48,378	33.38
Total	85,563	59.04

**Table 3:** The floral volatile organic compounds detected in *L. longituba* tepals.

No.	Name	S1-P	S2-P	S3-P
1	Benzoic acid	0.0018 ± 0.0007	4.5579 ± 0.5830	162.3274 ± 15.1509
2	E-2-Hexenyl benzoate	0.0032 ± 0.0007	-	-
3	Octanoic acid	0.0025 ± 0.0003	-	-
4	3-Methoxy-2,5-dimethylpyrazine	0.0053 ± 0.0018	-	-
5	Hydroxylamine	0.0016 ± 0.0004	-	-
6	7,9-Di-tert-butyl-1-oxaspiro(4,5) deca-6,9-diene-2,8-dione	0.0003 ± 0.0001	-	-
7	Hexadecanoic acid	0.0056 ± 0.0006	0.2546 ± 0.1015	-
8	1,3,6-Octatriene	-	13.0954 ± 5.7992	-
9	Benzenepropanoic acid	-	0.7522 ± 0.1997	1.5608 ± 0.5570
10	5-Amino-2-methoxy-4-picoline	-	0.2054 ± 0.0275	-
11	Octadecane	-	0.1720 ± 0.0195	-
12	Caryophyllene	-	0.1357 ± 0.0225	0.6197 ± 0.0981
13	Hexadecane	-	4.5644 ± 0.8763	12.3402 ± 1.8746
14	α-Farnesene	-	0.3350 ± 0.1492	-
15	Nonadecane	-	0.0984 ± 0.0116	-
16	1-Hexadecanol	-	0.0735 ± 0.0295	-
17	Heptacosane	-	0.2135 ± 0.0520	-
18	Butyl aldoxime	-	-	2.5247 ± 0.4620
19	*trans*-β-Ocimene	-	-	85.4689 ± 5.0140
20	Benzyl nitrile	-	-	10.2698 ± 2.6025
21	Benzoic acid	-	-	145.0866 ± 12.9669
22	1-Butanol	-	-	15.5708 ± 3.5003
23	β-Myrcene	-	-	1.4760 ± 0.2479
24	Eucalyptol	-	-	2.7087 ± 1.0158
25	2,4,6-Octatriene	-	-	2.6357 ± 0.8897
26	α-Terpineol	-	-	0.6584 ± 0.4979
27	3-Methoxy-5-methylphenol	-	-	0.6302 ± 0.1412
28	Benzene	-	-	1.9492 ± 0.4702
29	1,6,10-Dodecatrien-3-ol	-	-	0.0591 ± 0.0181

## Supplementary Materials

The following are available online https://www.mdpi.com/2223-7747/8/3/53/s1. Figure S1: GO classification of DEGs among different samples in *L. longituba*, Figure S2: The KEGG enrichment of DEGs among the different samples, Table S1: Species distribution of the BLAST hits for all homologous sequences, Table S2: Primers used for qRT-PCR.
