# Supplementary material for: Integrating Transcriptomic and GC-MS Metabolomic Analysis to Characterize Color and Aroma Formation during Tepal Development in Lycoris longituba
Source: Plants (Basel). 2019 Feb 28;8(3):53. doi: 10.3390/plants8030053 (PMC6473938; doi:10.3390/plants8030053)
Supplement: Supplementary file 1 [file plants-08-00053-s001.zip › Figure S2.docx]

**
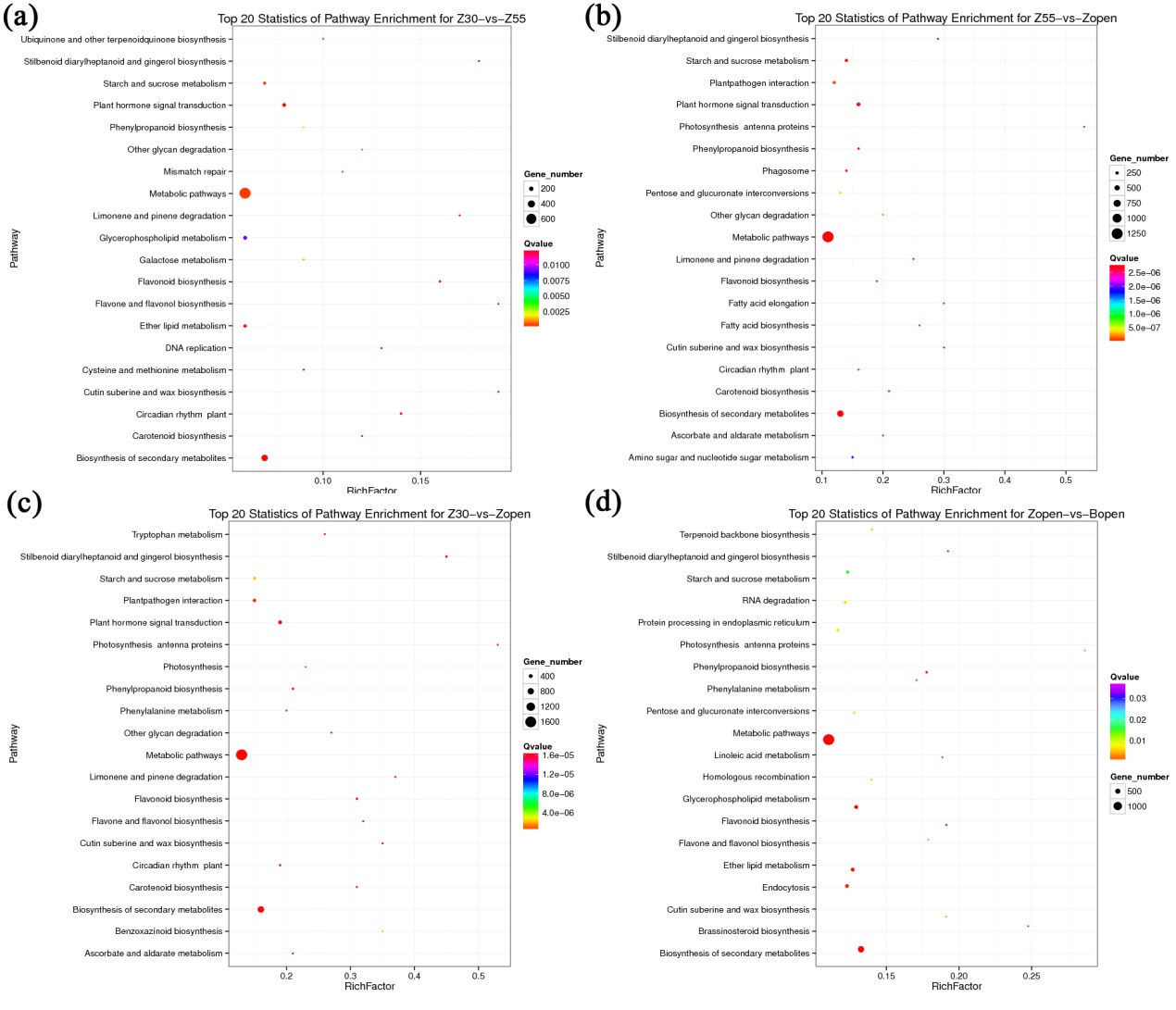
Figure S2.** The KEGG enrichment of DEGs among the different samples. The top 20 enriched KEGG pathways of DEGs of *L*. *longituba* tepals between S1-P and S2-P (a), S2-P and S3-P (b), S1-P and S3-P (c), as well as S1-P and S1-W (d).
