# Supplementary material for: Integrating Transcriptomic and GC-MS Metabolomic Analysis to Characterize Color and Aroma Formation during Tepal Development in Lycoris longituba
Source: Plants (Basel). 2019 Feb 28;8(3):53. doi: 10.3390/plants8030053 (PMC6473938; doi:10.3390/plants8030053)
Supplement: Supplementary file 1 [file plants-08-00053-s001.zip › Table S1.docx]

| **Table S1.** Species distributionof the BLAST hits for all homologous sequences. | |
| --- | --- |
| **Species** | **Distribution** |
| African oil palm | 29.60% |
| Phoenix dactylifera | 24.30% |
| Musa acuminata subsp. Malaccensis | 8.30% |
| Vitis vinifera | 3.00% |
| Indian lotus | 2.80% |
| Japanese rice | 1.50% |
| Zea mays | 1.20% |
| other | 29.20% |
