# Supplementary material for: Integrating Transcriptomic and GC-MS Metabolomic Analysis to Characterize Color and Aroma Formation during Tepal Development in Lycoris longituba
Source: Plants (Basel). 2019 Feb 28;8(3):53. doi: 10.3390/plants8030053 (PMC6473938; doi:10.3390/plants8030053)
Supplement: Supplementary file 1 [file plants-08-00053-s001.zip › Table S2.docx]

| **Table S2.** Primers used for qRT-PCR. | |
| --- | --- |
| Primer name | Primer sequence |
| LlPALa-qF | GGATAGTTATGGGGTGACGACG |
| LlPALa-qR | AGCGGATGCCCGAGTAGC |
| LlPALb-qF | CCCATGTTTCTTCCTCTACGC |
| LlPALb-qR | CACGCTTACCGCTGTCGC |
| LlCHSa-qF | CAAAGAGGCGGCAGTAAAGG |
| LlCHSa-qR | GCCGAGGAGTTTGGTGAGC |
| LlCHSb-qF | ATCACCGCCGTCACATTCC |
| LlCHSb-qR | TGCCCCTTCCGAGTCTGG |
| LlCHIa-qF | TCGGGGGTGCAGGTGTT |
| LlCHIa-qR | CGGTGTATTGTTCGCCAGTTAG |
| LlCHIb-qF | GTCCAAGGCTGTGTCTCAAGG |
| LlCHIb-qR | TTTCTTAAAACCTTTCATGGTACTCTC |
| LlF3Ha-qF | GGGAAATAGTGACCTACTTCTCATACC |
| LlF3Ha-qR | CCTCTGATAACACCTCCAGCAAC |
| LlF3Hb-qF | CCGCCAAATCAGTCCGC |
| LlF3Hb-qR | CTCCTTCAAATCCCCCCG |
| LlF3’Ha-qF | AAAGGGTGGATTCATCGTGTCT |
| LlF3’Ha-qR | AACCGTCGGGCTTGTCG |
| LlF3’Hb-qF | CCTACTCATACAACAGCAACAGCC |
| LlF3’Hb-qR | ATCAAGCACCCTATTCTCCACTC |
| LlF3’5’Ha-qF | GATGGTGAAGGGACACAGTGAGT |
| LlF3’5’Ha-qR | TTGTTGTGCCCGTTTTAGGAT |
| LlF3’5’Hb-qF | TAGGATGGGGGTTGTGATGGT |
| LlF3’5’Hb-qR | CCGTGGGGTAACCTTGGC |
| LlFLSa-qF | CGCAAATCTCAACACCATACCTT |
| LlFLSa-qR | CACAAGTTGAAAGATCCCCCAC |
| LlFLSb-qF | CCCACAAGCGGAGAAGGAG |
| LlFLSb-qR | ACCCAGCCCTTCTTCCCTT |
| LlDFRa-qF | ATCACTGCTAAAGACCACCAAGG |
| LlDFRa-qR | TGATAGCACATAAACCCATCCACT |
| LlDFRb-qF | AAGAACAAACTACTGTGACCAAAGC |
| LlDFRb-qR | CCATAAGCTTGTATTGTGGGTGT |
| LlDFRc-qF | GAAGTGATAAAGCCAGCAATAGACG |
| LlDFRc-qR | GATGATGTGAAAATAACTCGCTGG |
| LlANSa-qF | CCGTGGGCAAAGGGTTCT |
| LlANSa-qR | CCCGCAAGAGTTGTTCGC |
| LlLARa-qF | TCACCCTTGCGATGTTTGC |
| LlLARa-qR | TGCTCAGTTATGGTGACTCTTGG |
| LlUFGTa-qF | CGGCTTCATCCGTGGCT |
| LlUFGTa-qR | GTCTGCTCTGGCTCGGGTT |
| LlUFGTb-qF | GGTTTGTTGGTCCTGTTTCCC |
| LlUFGTb-qR | CCGAACCCTTTGGCTTAGACT |
