# Supplementary figures and images for: Integrating Transcriptomic and GC-MS Metabolomic Analysis to Characterize Color and Aroma Formation during Tepal Development in Lycoris longituba
Source: Plants (Basel). 2019 Feb 28;8(3):53. doi: 10.3390/plants8030053 (PMC6473938; doi:10.3390/plants8030053)

**Figure S1.** GO classification of DEGs among different samples in *L*. *longituba*.


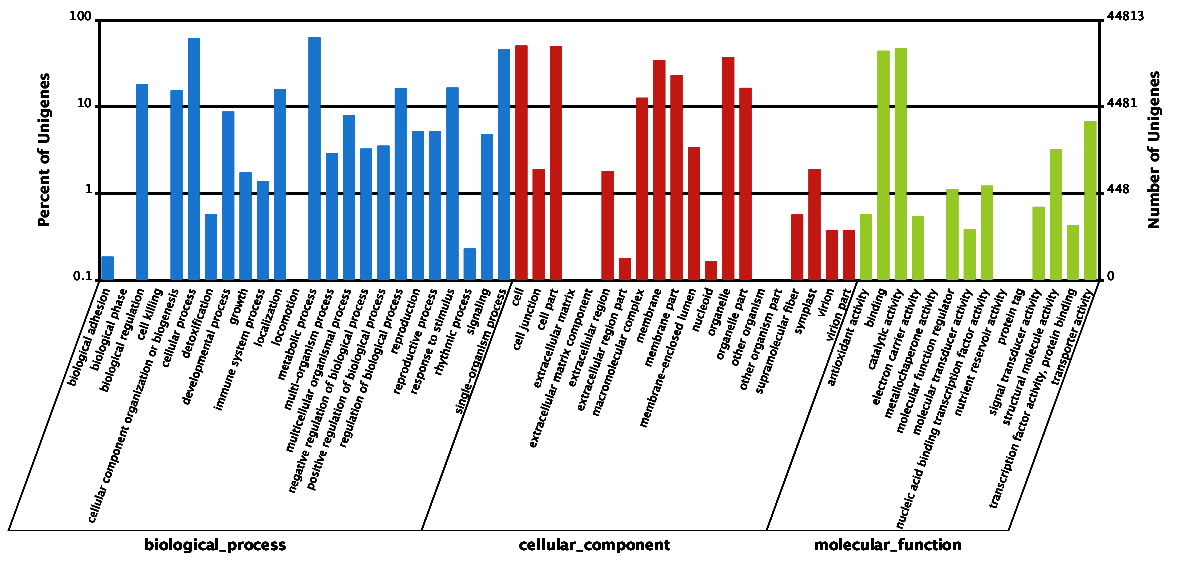

Supplement: Supplementary file 1 [file plants-08-00053-s001.zip › Figure S1.docx]
